# The *Neospora caninum* Paradox: Comparative Biology of Cattle and Water Buffalo Reveals Pathways to Control Bovine Neosporosis

**DOI:** 10.3390/microorganisms14061329

**Published:** 2026-06-13

**Authors:** Chiara Storoni, Anna-Rita Attili, Michael Okoli, Yubao Li, Vincenzo Cuteri

**Affiliations:** 1 School of Biosciences and Veterinary Medicine, University of Camerino, 62024 Matelica, Italy; chiara.storoni@unicam.it (C.S.); annarita.attili@unicam.it (A.-R.A.); 2 School of Medicine & Veterinary Medicine, Trakia University, 6000 Stara Zagora, Bulgaria; michael.okoli.22@trakia-uni.bg; 3 School of Pharmaceutical Sciences and Food Engineering, Liaocheng University, Liaocheng 252000, China; liyubao@lcu.edu.cn

**Keywords:** *Neospora caninum*, cattle, buffalo, abortion, vaccine, *Toxoplasma gondii*

## Abstract

*Neospora caninum* is a major cause of reproductive failure in cattle, responsible for epidemic abortion outbreaks that inflict annual billion-dollar losses on the global livestock industry. In water buffaloes (*Bubalus bubalis*), however, a phylogenetically close relative often raised in the same environments, the same parasite typically establishes a subclinical persistent infection with markedly lower rates of clinical abortion. This review inverts the traditional narrative by arguing that the key to next-generation control strategies lies in understanding the tolerant host (buffalo) rather than solely the susceptible host (cattle). By dissecting this “*Neospora* paradox”, we explore the molecular and immunological crosstalk that dictates pregnancy outcomes. We examine the parasite’s invasion proteins, revealed by CRISPR-Cas9 screens, and the maternal–fetal interface, where the balance between immune tolerance and parasite control determines the fate of pregnancy. We also compare *N. caninum* with the related zoonotic parasite *Toxoplasma gondii* to highlight how differential host immune recognition shapes infection outcomes. Finally, we propose that deciphering the buffalo’s successful equilibrium with *N. caninum* can illuminate novel pathways for vaccines and immunotherapeutic strategies, transforming the management of neosporosis worldwide.

## 1. Introduction

### 1.1. The Economic and Reproductive Burden of Bovine Neosporosis

Neosporosis is the most frequently diagnosed cause of bovine abortion worldwide, representing a formidable challenge to global livestock productivity [1]. The economic toll is staggering: conservative estimates place annual losses to the global cattle industry at approximately $1.3 billion, arising from direct abortion losses, premature culling of infected animals, reduced milk production, and diminished genetic progress [2]. In endemic regions, seroprevalence rates in dairy herds can exceed 80%, and epidemic abortion outbreaks can eliminate 10–30% of a season’s calf crop within weeks [3].

### 1.2. The Water Buffalo: An Unexpectedly Tolerant Host

In contrast, water buffaloes (*Bubalus bubalis*) present a fundamentally different clinical picture, despite sharing pastures, management systems, and geographic regions with cattle across Asia, the Mediterranean, and parts of South America. Extensive seroepidemiologic surveys reveal that buffaloes are frequently exposed to *N. caninum*—seroprevalence rates often equal or exceed those in co-located cattle populations. A comprehensive review of studies from Iran reported seroprevalence ranging from 19.2% to 55.9% in water buffaloes [4,5]. Similar findings emerge from Italy [6], Brazil [7], and Egypt [8]. Yet, despite this high exposure, associated reproductive pathology is remarkably muted. Clinical abortion outbreaks in buffalo herds are rare, and when they occur, *N. caninum* is seldom identified as the primary etiological agent [9].

This presents the central paradox: why does the same parasite produce devastating disease in one closely related ruminant species while establishing a seemingly benign coexistence in another? This review argues that comparative biology—contrasting the susceptible bovine host with the tolerant buffalo host—represents a powerful and underutilized tool to unravel the complex pathophysiology of neosporosis and forge new paths for intervention. By treating the buffalo as the central subject of inquiry, we can identify the immunological and molecular circuit breakers that prevent disease and translate these discoveries into actionable control strategies for cattle.

## 2. *Neospora caninum*: Life Cycle, Transmission, and Pathogenesis in Traditional Hosts

### 2.1. The Life Cycle

*Neospora caninum* is an obligate intracellular apicomplexan parasite with a heteroxenous life cycle requiring both definitive and intermediate hosts [10]. Canids—domestic dogs (*Canis lupus familiaris*), coyotes (*Canis latrans*), and gray wolves (*Canis lupus*)—serve as definitive hosts, shedding environmentally resistant oocysts in their feces [1,11,12]. Intermediate hosts, primarily cattle and other ruminants, become infected through ingestion of sporulated oocysts contaminating feed or water.

Within the intermediate host, the parasite undergoes a biphasic developmental program. Following ingestion, sporozoites excyst in the intestine, differentiate into rapidly dividing tachyzoites, and disseminate via the bloodstream throughout the host [13]. This acute phase is characterized by widespread parasite proliferation and active invasion of nucleated cells. In immunocompetent hosts, the immune response—particularly interferon-gamma (IFN-γ)-mediated mechanisms—drives tachyzoite conversion into slowly dividing bradyzoites, which form tissue cysts predominantly within neural and muscular tissues [14]. These latent cysts persist for the lifetime of the host, representing a reservoir for potential recrudescence and a vehicle for vertical transmission.

#### Definitive Hosts and Geographic Considerations

The efficiency of horizontal transmission depends on definitive host density and behavior. Domestic dogs are the most relevant definitive hosts in agricultural settings worldwide [11]. Coyotes and wolves contribute to sylvatic cycles in North America and Europe [12,15]. Critically for the *Neospora* paradox, definitive host populations do not differ systematically between cattle- and buffalo-raising regions. In many areas—including the Mediterranean (Italy, Egypt), South America (Brazil, Venezuela), and Asia (India, Pakistan, China)—cattle and buffalo share pastures, water sources, and exposure to the same canine populations. Therefore, differential exposure to oocysts is unlikely to explain the divergent clinical outcomes, strengthening the hypothesis that host-specific factors underlie the paradox (Supplementary Materials S1).

### 2.2. Transmission Dynamics

*Neospora caninum* employs two transmission strategies, each with distinct epidemiological implications. Horizontal transmission occurs when susceptible animals ingest oocysts shed by definitive hosts [16]. While this route maintains environmental contamination and introduces infection into naïve herds, its efficiency is limited by several factors: oocyst shedding by dogs is often transient, environmental oocyst survival is variable, and the infectious dose required to establish infection remains poorly characterized [17].

Vertical (transplacental) transmission represents the parasite’s evolutionary masterstroke for population persistence [18]. This route occurs in two contexts. Exogenous transplacental transmission results from primary maternal infection acquired during pregnancy, wherein tachyzoites cross the placenta and infect the fetus [18]. Exogenous transmission—typically following ingestion of oocysts from contaminated feed or water—is considered the major driver of epidemic abortion outbreaks in cattle herds [18,19]. Endogenous transplacental transmission arises from recrudescence of a chronic infection: pregnancy-associated immunomodulation triggers bradyzoite reactivation to tachyzoites, which then cross the placenta [20]. This endogenous route ensures perpetuation of infection across generations, with infected heifers giving birth to infected calves that maintain the parasite within the herd indefinitely. Indeed, vertical transmission efficiency can approach 95% in persistently infected cows (primarily via endogenous transmission), making neosporosis a quintessential example of a vertically maintained infection [21].

### 2.3. The Clinical Picture in Cattle

The pathological consequences of *N. caninum* infection in cattle are most dramatically manifested during gestation. Abortion typically occurs between 4 and 6 months of gestation, with the peak incidence at 5–6 months (mid to late gestation) [20,22]. The pathogenesis involves multifocal placentitis with necrosis of cotyledonary villi, followed by fetal infection and systemic disease. Affected fetuses exhibit nonsuppurative encephalitis characterized by perivascular cuffing, multifocal gliosis, and necrosis—often with detectable tissue cysts in the brain [23]. Myocarditis, hepatitis, and myositis are also commonly observed [1].

The immunopathological basis of abortion lies in the delicate balance required for successful pregnancy. The maternal–fetal interface represents an immunological paradox: the semi-allogeneic fetus must be protected from maternal immune rejection while maintaining the capacity to combat pathogens. This is achieved through a Th2-biased immunological environment, characterized by anti-inflammatory cytokines including IL-4, IL-5, and IL-10 [24]. However, *N. caninum* is an intracellular pathogen requiring Th1-type immunity—particularly IFN-γ—for control [25]. Some immune adaptations during pregnancy may favor parasite survival and transplacental transmission. The pregnancy-induced shift away from Th1 responses creates a permissive environment for tachyzoite proliferation and transplacental invasion.

## 3. The *Neospora caninum* Paradox: Contrasting Outcomes in Cattle and Buffalo

### 3.1. Epidemiological Evidence

The contrasting outcomes of *N. caninum* infection in cattle and buffaloes are well documented across diverse geographic settings. A meta-analysis of global seroprevalence studies confirms that, while exposure rates are comparable between the two species, abortion risk diverges dramatically [26]. In Iran, a survey of 236 water buffaloes from one province revealed an overall seroprevalence of 17.7% using a commercial ELISA, yet follow-up investigations failed to establish *N. caninum* as a significant cause of buffalo abortion [27]. Similarly, in southern Italy—a region with intensive buffalo dairy production—Guarino et al. [6] reported 29.8% seropositivity in 1547 buffalo samples, but retrospective analysis of abortion cases over a 5-year period identified *N. caninum* in less than 2% of fetal examinations.

The pattern extends to South America. In Brazil, where both cattle and buffaloes are extensively raised, seroprevalence in buffaloes ranges from 22.7% to 65.5% across different states [7,28]. Yet worldwide, seroprevalence of *N. caninum* infection in buffaloes is high, at approximately 48%—three to four times higher than that reported in the world’s cattle populations (16.1% for dairy cattle and 11.5% for beef cattle)—despite clinical manifestations such as abortion being rare in buffaloes [26]. Indeed, while *N. caninum* is considered one of the major causes of abortion in cattle; its reproductive importance is apparently lower in buffaloes [29].

However, direct comparisons between cattle and buffalo must be interpreted with caution. Seroprevalence studies in buffalo vary considerably in methodology, sample size, and geographic scope, and abortion events in buffalo may be underreported—particularly in less developed regions where monitoring is limited. Additionally, buffaloes generally have lower reproductive efficiency and seasonal breeding patterns, which may make reproductive losses less noticeable compared to cattle [26]. These factors do not negate paradoxical observation but place it in an appropriate context.

### 3.2. Beyond Seroprevalence: What Is Happening at the Placental Level?

Experimental infection studies have provided crucial insights into the mechanistic basis of the buffalo’s reduced clinical susceptibility. Konrad et al. [30] experimentally infected pregnant buffalo heifers with *N. caninum* tachyzoites and monitored outcomes through gestation. Despite confirmed seroconversion and detection of parasite DNA in maternal blood, only two of 12 fetuses showed histological lesions consistent with neosporosis, and these were mild compared to the severe encephalitis typical of bovine infections. Importantly, parasite transmission to the fetuses occurred (six of 12 fetuses were PCR-positive), but this transmission was not associated with fetal death or significant pathology.

This dissociation between parasite transmission and disease is the critical observation. In cattle, vertical transmission does not invariably lead to abortion; the outcome depends on multiple factors, including the stage of gestation (infections later in gestation often result in live, persistently infected calves), parasite isolate virulence, and maternal immune status [14,18]. However, when transmission occurs during mid-gestation (4–6 months, corresponding to the second trimester)—the period of peak susceptibility in cattle—it is frequently associated with elevated abortion risk [17]. In buffaloes, by contrast, transmission can occur without triggering the inflammatory cascade that causes fetal death, even during mid-gestation [30]. Additionally, gestation length differs between species (buffalo: 310–315 days; cattle: 280–285 days), which may affect the timing and consequences of transplacental infection relative to fetal immune development. This interspecies difference has not been systematically investigated and represents a gap in current knowledge.

**Figure 1:**
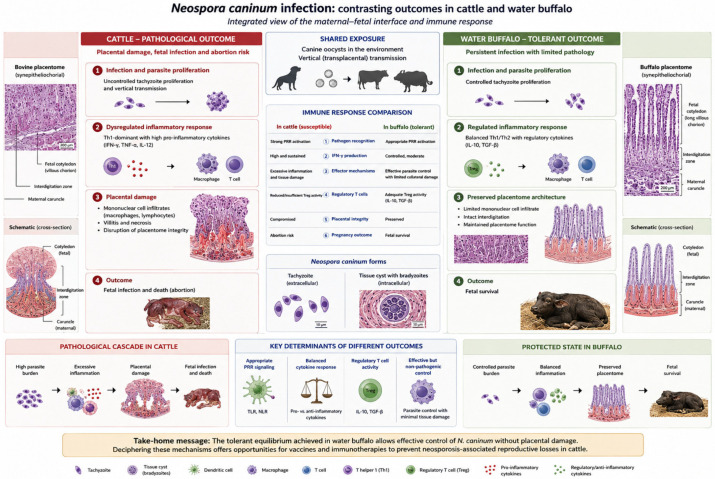
Integrated comparison of placental architecture, immune responses, and clinical outcomes following *Neospora caninum* infection in cattle and water buffalo. Both species are exposed to similar sources of infection, including environmental contamination with canid-derived oocysts and vertical (transplacental) transmission. In cattle (**left panels**), uncontrolled tachyzoite proliferation is associated with a predominantly Th1-biased inflammatory response characterized by increased IFN-γ, TNF-α, and IL-12 production; mononuclear inflammatory infiltrates (mainly macrophages and T lymphocytes), villitis, placental necrosis, and disruption of placentome integrity, ultimately leading to fetal infection and abortion. In contrast, water buffalo (**right panels**) exhibit a more regulated and balanced immune response, with increased regulatory cytokines (IL-10 and TGF-β), preserved synepitheliochorial placentome architecture, limited inflammatory damage, and a higher likelihood of fetal survival despite persistent infection. Representative histological and schematic placentome sections illustrate species-specific morphological differences. Cattle placentomes show broader interdigitated villous structures, whereas buffalo placentomes are characterized by longer and more slender villi. The central panels summarize the proposed mechanisms underlying the “tolerant equilibrium” observed in buffalo, including regulated cytokine responses, enhanced regulatory T cell activity, preservation of placental integrity, and improved control of parasite proliferation with limited collateral tissue damage. Parasite forms are represented separately as extracellular tachyzoites and intracellular tissue cysts containing bradyzoites. Abbreviations: PRR, pattern recognition receptor; TLR, Toll-like receptor; NLR, NOD-like receptor; Treg, regulatory T cell; Th1, T helper 1 cell; IFN-γ, interferon-γ; TNF-α, tumor necrosis factor-α; IL, interleukin.

Ueno et al. [31] provided histological evidence supporting this hypothesis. Comparative examination of placentomes from naturally infected cattle and buffaloes revealed striking differences. Bovine placentomes exhibited extensive necrosis, mononuclear inflammatory infiltration, and edema—lesions that compromise fetal nutrient and gas exchange. Buffalo placentomes, by contrast, showed minimal inflammatory changes despite the presence of parasite DNA in adjacent tissues. The architecture of the placentome remained largely intact, suggesting that the buffalo placenta either restricts parasite invasion or mounts a regulated inflammatory response that limits collateral tissue damage (Figure 1).

### 3.3. A Question of Co-Evolution

These observations raise a compelling evolutionary hypothesis: has *N. caninum* enjoyed a longer co-evolutionary history with *Bubalus bubalis*, leading to a more balanced host–parasite relationship? Or have buffaloes evolved unique immunological “circuit breakers” that prevent the uncontrolled tachyzoite proliferation and inflammatory pathology seen in cattle?

The water buffalo’s evolutionary trajectory diverged from that of taurine cattle approximately 3–5 million years ago [32]. *N. caninum* likely co-evolved with canid definitive hosts and wild ruminant intermediate hosts long before domestication [33]. If buffaloes served as ancient intermediate hosts in regions where the parasite was endemic, natural selection would have favored alleles conferring reduced pathology while maintaining transmission competence [34]. This “domestication” of the parasite—the evolution of tolerance rather than resistance—would manifest as the silent infections observed today. Cattle, by contrast, may represent a more recent host, having expanded into parasite-endemic regions during human-mediated migrations without the benefit of millennia of co-adaptive evolution. The result could be a maladapted host–parasite relationship characterized by excessive inflammation, tissue destruction, and reproductive failure.

## 4. Molecular Mechanisms of Host–Parasite Interactions

### 4.1. The Parasite’s Toolkit

Understanding the buffalo’s protective mechanisms requires an appreciation of the parasite’s sophisticated invasion machinery. *N. caninum* shares with other apicomplexans a specialized set of apical organelles—micronemes, rhoptries, and dense granules—that sequentially discharge their contents during host cell invasion [35].

**Microneme proteins (MICs)** initiate contact with the host cell. These adhesins, including NcMIC1, NcMIC3, and NcMIC6, mediate parasite attachment to host cell surface receptors and are essential for gliding motility [36]. Structural studies reveal that NcMIC1 contains microneme adhesive repeat (MAR) domains that bind sialic acid residues on host cell surfaces, a mechanism conserved with *T. gondii* [37]. Antibodies targeting MICs can inhibit invasion in vitro, highlighting their vulnerability as vaccine targets [38].

**Rhoptry proteins (ROPs)** are discharged following microneme-mediated attachment and play dual roles in invasion and modulation of host cell functions. ROPs are injected into the host cell cytoplasm, where they localize to the parasitophorous vacuole membrane (PVM) or traffic to the host cell nucleus [39]. NcROP40, a pseudokinase localized to the rhoptry bulbs, is more abundantly expressed in virulent isolates and has been implicated as a potential virulence factor involved in the manipulation of innate immune defense mechanisms [40,41]. NcROP16 traffics to the nucleus and modulates host gene expression, potentially creating a more permissive intracellular environment [42].

**Dense granule proteins (GRAs)** are secreted throughout intracellular development and contribute to the modification of the parasitophorous vacuole. NcGRA6 and NcGRA7 localize to the PVM and vacuolar lumen, where they participate in nutrient acquisition and immune evasion [43]. NcGRA7 is particularly immunogenic and has been exploited for diagnostic applications [44]. Notably, GRAs from *N. caninum* and *T. gondii* exhibit only 40–60% amino acid identity, suggesting species-specific adaptations to host environments.

### 4.2. CRISPR-Cas9 and Virulence

The application of CRISPR-Cas9 gene editing to apicomplexan parasites has revolutionized the understanding of *N. caninum* virulence factors. Research has applied CRISPR-Cas9 to dissect the function of specific *N. caninum* genes. For instance, NcROP5 has been identified as a key virulence factor, as its deletion significantly attenuates virulence in a murine model [45]. Similarly, the generation of knockout mutants for NcGRA7 and NcROP40 has revealed their involvement in parasite virulence, with NcGRA7 deletion considerably impairing pathogenicity in pregnant mice [46]. The application of CRISPR-Cas9 in *N. caninum* has been further advanced by the development of TaqMan-quantitative PCR assays to reliably assess mutagenesis efficiency [47].

Using this approach, Yang et al. [48] identified the dense granule protein NcGRA17 as an important regulator of parasitophorous vacuole morphology and pathogenicity, with knockout strains exhibiting aberrant PV formation and loss of virulence. Similarly, Du et al. [49] generated NcMYR1 knockout strains, demonstrating that NcMYR1 is a virulence factor critical for processes such as invasion and replication.

It is important to note, however, that there is currently limited evidence supporting host-specific adaptation of *N. caninum* strains to cattle versus buffalo. The proposal that isolates from these hosts may differ genetically or in effector expression remains hypothetical and requires direct investigation. The value of CRISPR-Cas9 tools lies in their potential to test such hypotheses, not in established knowledge of host adaptation.

### 4.3. The Immunological Conflict at the Maternal–Fetal Interface

The host immune response to *N. caninum* represents a double-edged sword, particularly during pregnancy. The **Th1/Th2 paradigm** provides the conceptual framework: successful pregnancy requires a Th2-biased uterine environment dominated by IL-4, IL-5, and IL-10, which suppresses cell-mediated immunity and promotes maternal-fetal tolerance [50]. However, control of intracellular pathogens like *N. caninum* depends on Th1 immunity, particularly IFN-γ production by NK cells and CD4+ T lymphocytes [51]. IFN-γ activates macrophages to kill intracellular parasites, induces nitric oxide production, and promotes Th1 polarization.

This immunological conflict creates vulnerability. During mid-gestation—coinciding with peak *N. caninum* abortion risk—placental IFN-γ expression is naturally downregulated to protect the fetus [20]. This creates a window of opportunity for tachyzoite recrudescence and transplacental invasion. Experimental studies in murine models confirm that neutralization of IFN-γ dramatically increases vertical transmission and fetal pathology [50,52]. While direct IFN-γ neutralization experiments have not been reported in pregnant cattle, correlative studies show that reduced IFN-γ bioavailability at the maternal–fetal interface is associated with increased parasite recrudescence and transplacental transmission [20,52].

**Recrudescence** represents the practical manifestation of this immunological conflict. Latent bradyzoite cysts persist within neural tissues, continuously monitored by the immune system. Pregnancy-induced immunomodulation—particularly reduced IFN-γ bioavailability—can trigger bradyzoite-to-tachyzoite conversion [53]. Released tachyzoites then disseminate, reaching the placenta and fetus. This endogenous transplacental transmission explains how chronically infected cows can abort multiple times or produce infected offspring across successive pregnancies.

#### Epigenetic Regulation of Stage Conversion

Beyond cytokine signaling, epigenetic mechanisms are increasingly recognized as key regulators of the bradyzoite-tachyzoite switch. In *T. gondii*, histone acetyltransferases (e.g., GCN5) and deacetylases (e.g., HDAC3) control the expression of stage-specific genes [52,54]. While similar chromatin-modifying machinery is predicted to be conserved in *N. caninum* based on genomic analysis [55], direct experimental evidence in *N. caninum* remains limited. Host-mediated epigenetic modifications—such as DNA methylation of parasite promoters or changes in host cell chromatin accessibility during pregnancy—could influence recrudescence efficiency, but this hypothesis requires experimental testing.

### 4.4. The Buffalo Difference: Hypotheses

The buffalo’s reduced susceptibility to *N. caninum*-induced abortion likely reflects modifications to the immunological balancing act described above. Several non-mutually exclusive hypotheses warrant investigation.

**Hypothesis** **1.** ***Placentomal structural differences.** The buffalo placenta exhibits distinct morphological features compared to cattle, including non-stalked placentomes with long, slender, and moderately branched villi, in contrast to the stalked, mushroom-shaped placentomes with broad, conical, and complexly branched villi observed in cattle [56]. Additionally, the fetal vasculature of the water buffalo placentome undergoes progressive development throughout pregnancy, with conical villous trees changing from wide to slender shapes as gestation advances [57]. These structural differences might physically impede tachyzoite invasion or restrict dissemination within placental tissues. The observation of intact buffalo placentome architecture despite parasite presence [31] supports this mechanical barrier hypothesis*.

**Hypothesis** **2.** ***Enhanced local immune regulation.** Buffaloes may possess more abundant or functionally enhanced regulatory T cell (Treg) populations at the maternal–fetal interface. Tregs suppress effector T cell responses through IL-10 and TGF-β production, potentially limiting both anti-parasite immunity and inflammatory pathology [58]. If the Treg cells at the buffalo’s maternal–fetal interface were more numerous or functionally more effective, they might limit the inflammation caused by the parasite, whilst maintaining sufficient control over the parasite to prevent a severe infection*.

**Hypothesis** **3.** ***More stringent control of parasite recrudescence.** The balance between bradyzoite latency and reactivation is influenced by host immune status and parasite-intrinsic factors. Buffaloes might maintain higher baseline IFN-γ levels in neural tissues where cysts reside, preventing recrudescence [20,52]. Alternatively, buffalo bradyzoites might exhibit intrinsically lower reactivation rates due to host-imposed mechanisms*.

**Hypothesis** **4.** ***Distinct cytokine response profiles.** Comparative transcriptomic analyses could reveal species-specific differences in the kinetics or magnitude of cytokine responses. Perhaps buffaloes mount rapid, localized IFN-γ responses sufficient to control tachyzoites without systemic Th1 polarization that threatens pregnancy [59]. This “Goldilocks” response—neither too weak (allowing uncontrolled parasite growth) nor too strong (causing immunopathology)—would represent the ideal equilibrium*.

**Hypothesis** **5.** ***Genetic polymorphisms in innate immune receptors.** Toll-like receptors (TLRs) and MHC class I/II molecules exhibit species-specific polymorphisms that shape pathogen recognition and antigen presentation. Genome-wide association studies (GWAS) comparing infected cattle and buffaloes might identify buffalo alleles associated with protection. Candidate genes include TLR2 and TLR4, which recognize parasite glycosylphosphatidylinositol anchors [60], and MHC class II genes that determine immunodominant epitope presentation*.

### 4.5. The Parasite Perspective: Do N. caninum Isolates Differ Between Host Species?

The preceding sections have focused primarily on host factors that may explain the contrasting outcomes of *N. caninum* infection in cattle and buffalo. However, the parasite itself may contribute to this paradox. If *N. caninum* strains circulating in cattle differ genetically or phenotypically from those infecting buffalo, observed differences in clinical outcome could reflect parasite adaptation rather than—or in addition to—host factors.

*Current knowledge of isolate diversity*: Molecular epidemiological studies have identified significant genetic diversity among *N. caninum* isolates globally, with microsatellite and multilocus sequencing analyses revealing distinct strain lineages [61,62]. However, virtually all characterized isolates originate from cattle, dogs, or laboratory passage. Laboratory passage refers to serial in vitro cultivation of isolates in cell culture or serial passage through immunocompromised mice. This process may select for laboratory-adapted phenotypes that do not fully reflect virulence in natural hosts. Isolates with extensive passage history (e.g., Nc-1) should be interpreted with this caveat. To date, no *N. caninum* isolate has been definitively characterized from water buffalo.

*Virulence differences among characterized isolates*: Experimentally, *N. caninum* isolates show marked variation in virulence in murine and bovine models. The Nc-1 isolate (isolated from a dog in the USA) is generally considered to display moderate virulence, with abortion rates in experimentally infected cattle ranging from 30 to 60% depending on challenge dose and gestational stage [10,63]. The Nc-Spain-7 isolate (isolated from cattle in Spain, where multiple isolates with differing virulence profiles exist) exhibits higher virulence in pregnant mouse models, while other Spanish isolates (e.g., Nc-Spain-1H) show attenuated phenotypes [63]. Nc-Nowra (isolated from a naturally infected subclinical calf in Australia) shows reduced virulence and has been evaluated as a live vaccine candidate [64]. Nc-Argentina LP1 (isolated from a naturally infected calf in Argentina) has been characterized as a low-virulence isolate, though detailed bovine challenge studies are pending [65].

Critically, experimental infections using the same *N. caninum* isolate in different host species are particularly informative for distinguishing host versus parasite contributions to clinical outcomes. Konrad et al. [30] used the Nc-1 isolate to experimentally infect both pregnant cattle and water buffalo heifers under comparable conditions. While cattle developed severe placentitis and fetal death, buffalo heifers showed minimal pathology despite confirmed vertical transmission—directly demonstrating that host factors, rather than parasite strain differences, are sufficient to explain the divergent outcomes. This finding does not exclude the possibility that additional adaptation may occur, but establishes that the paradox exists even with identical parasite isolates.

*A critical knowledge gap*: It must be acknowledged that all currently characterized *N. caninum* isolates—including Nc-1, Nc-Liverpool, Nc-Spain7, Nc-Spain1H, Nc-Nowra, and Argentine isolates—originate from cattle, dogs, or laboratory passage. No isolate has yet been obtained from naturally infected water buffalo. Therefore, while experimental cross-infection studies using the same isolate [30] unequivocally demonstrate that host factors are sufficient to explain the cattle-buffalo paradox, the possibility that field strains circulating in each host species may have undergone host-specific adaptation cannot be excluded. Genetic diversity among *N. caninum* isolates is well documented [61,62], and strain-specific differences in virulence-associated effectors (e.g., ROP5, ROP16, GRA7) could theoretically influence clinical outcomes in a host-dependent manner. The absence of buffalo-derived isolates remains a critical gap that prevents definitive assessment of this possibility. Future work must prioritize the isolation, genotyping, and experimental characterization of *N. caninum* from naturally infected water buffalo across multiple geographic regions to determine whether host-adapted strains contribute to the tolerant phenotype observed in this species.

*Hypothesis deserving investigation*: It remains plausible that buffalo-adapted strains—if they exist—may differ in virulence, but the experimental evidence indicates that host factors are the primary determinants of the differential phenotype. Future work should prioritize: (1) isolation and genomic characterization of *N. caninum* from naturally infected buffalo across multiple geographic regions; (2) comparative experimental infections using cattle-derived versus buffalo-derived isolates; and (3) genomic analyses to identify strain-specific polymorphisms in virulence-associated effectors.

Table 1 summarizes currently characterized *N. caninum* isolates with known virulence phenotypes.

## 5. New Horizons in Diagnostics and Control: Learning from the Tolerant Host

The previous sections have dissected the mechanistic basis of the *Neospora* paradox. We now turn to translational applications: how understanding the buffalo’s successful equilibrium can guide novel diagnostics, vaccines, and therapeutics for cattle.

### 5.1. Learning from the Buffalo: Biomarkers of Protection

The identification of protective immune profiles associated with successful pregnancy outcomes in infected buffaloes represents a distinct objective from the routine diagnosis of infection. Rather than reviewing diagnostic methods extensively, we focus here on how comparative approaches could reveal biomarkers of protection.

Conventional serodiagnosis of *N. caninum* infection relies on ELISA, IFAT, and immunoblotting using tachyzoite antigens (e.g., NcSAG1, NcGRA7) [66,67]. While these tools effectively detect exposure, they do not predict pregnancy outcome. The critical question for translating the buffalo paradox into practice is: can we identify serological or cellular biomarkers that distinguish “tolerant” from “susceptible” infection states?

*Implication from the buffalo paradox*: Comparative transcriptomic and proteomic analyses of infected cattle and buffalo placentomes could reveal species-specific expression signatures associated with protection. Candidate biomarkers might include: (1) regulatory T cell-associated cytokines (IL-10, TGF-β); (2) ratios of Th1 to Th2/Th17 responses at the maternal–fetal interface; and (3) specific miRNA profiles that regulate inflammatory signaling. Such biomarkers, once validated, could inform prognosis in infected cattle and serve as endpoints for vaccine trials [68].

### 5.2. Vaccines Development: Lessons from Attenuated Isolates

The quest for an effective *N. caninum* vaccine has spanned three decades with limited success. Traditional approaches—killed whole parasites, live-attenuated strains, and subunit vaccines—have each encountered obstacles [69].

Killed vaccines (e.g., Bovilis^®^ Neoguard, now withdrawn from the market) showed initial promise in reducing abortion risk but provided incomplete protection and required frequent boosting [70]. Live-attenuated vaccines generated through prolonged in vitro passage induce stronger cellular immunity but raise safety concerns regarding reversion to virulence and residual pathogenicity in immunocompromised animals [71]. The Nc-Nowra isolate (Australia), characterized by naturally attenuated virulence, has been evaluated as a live vaccine candidate in mice [72] and cattle [64]. Immunization with Nc-Nowra prior to challenge with virulent isolates (e.g., Nc-Liverpool or Nc-1) reduced vertical transmission and fetal pathology in experimental challenge models [64,69]. Similarly, the Nc-Spain-1H isolate (Spain), a naturally avirulent strain identified from seropositive but clinically normal cattle, has been evaluated in murine models and shown to induce protective immunity without causing abortion [63].

However, translation to field conditions has faced challenges. Killed whole tachyzoite vaccines, despite inducing antibody responses, have generally failed to prevent transplacental transmission in pregnant cattle following experimental challenge [70,73]. This limitation reflects the central immunological challenge: effective protection requires cell-mediated Th1 immunity, which conflicts with the pregnancy-associated Th2 bias.

The buffalo’s successful equilibrium suggests an alternative immunomodulatory approach. Rather than aiming for sterile immunity (which may require pro-inflammatory responses that threaten pregnancy), vaccines could be designed to induce “tolerant-protective” profiles characterized by:

− Controlled IFN-γ responses sufficient to limit tachyzoite proliferation
− Concurrent induction of regulatory T cell activity (IL-10, TGF-β)
− Preservation of placental integrity

Several research groups are exploring subunit vaccines targeting invasion proteins (MICs, ROPs) combined with adjuvants that promote balanced Th1/Treg responses [69].

*Implication from the buffalo paradox*: The tolerant buffalo phenotype suggests that an effective vaccine need not achieve sterile immunity; instead, it could aim to recapitulate the “Goldilocks” state—controlled parasite replication without inflammatory pathology. Achieving this may require adjuvants that induce balanced Th1/Treg responses, or the inclusion of buffalo-derived protective antigens identified through comparative screening.

### 5.3. Therapeutics

Pharmacological intervention for neosporosis remains limited, particularly in food animals where tissue residue concerns restrict drug options. Repurposed compounds with in vitro activity against *N. caninum* include:

− Monensin, an ionophore antibiotic widely used as a ruminant feed additive, inhibits *N. caninum* proliferation in cell culture at micromolar concentrations [74]. However, field trials evaluating monensin for prevention of bovine neosporosis have shown inconsistent results, and no chemoprophylactic agent has demonstrated reliable efficacy under field conditions [74,75].
− Pyrimethamine and sulfadiazine, synergistic inhibitors of folate metabolism, effectively control acute toxoplasmosis in humans and have demonstrated activity against *N. caninum* in vitro [75]. However, their use in cattle is precluded by long withdrawal times for milk and meat, and concerns about teratogenicity during pregnancy.
− Toltrazuril, a triazine derivative used for coccidiosis control, inhibits *N. caninum* replication in vitro and has shown efficacy in murine and experimental ruminant models, including pregnant sheep. It has also been evaluated under field conditions in cattle, where treatment was associated with reduced abortion rates; however, these findings derive from observational studies without a randomized controlled design and should therefore be interpreted with caution [76,77,78].
− Niclosamide has recently emerged as a promising therapeutic candidate, acting through NLRP3 inflammasome activation and direct antiparasitic effects that reduce invasion, intracellular proliferation, and mitochondrial function in tachyzoites, while improving survival and reducing tissue damage in infected mice [79]. This drug has yet to be evaluated in cattle and buffalo.

*Implication from the buffalo paradox*: Identification of buffalo-specific protective pathways (e.g., enhanced Treg function, distinct cytokine profiles) could lead to immunomodulatory therapies that induce a similar tolerant state in cattle, without the need for direct antiparasitic drugs.

### 5.4. Practical Field Management Implications

While buffalo-derived biomarkers represent future directions, current management of bovine neosporosis relies on integrated control measures: (1) preventing farm dogs from accessing aborted fetuses or placental tissues; (2) providing clean feed and water sources not contaminated by dog feces; (3) culling chronically infected cows with repeated abortion events; (4) maintaining closed herds or testing introduced animals; and (5) implementing biosecurity protocols to limit wildlife access to livestock areas. These measures reduce but do not eliminate transmission, underscoring the need for the novel approaches proposed herein.

## 6. Comparative Biology of *Neospora caninum* and *Toxoplasma gondii*: Lessons in Host Susceptibility

The comparison between *N. caninum* and *T. gondii* is particularly informative when framed around patterns of host susceptibility. *T. gondii* infects a remarkably broad range of intermediate hosts, including cattle, but with an important nuance: while seroprevalence in cattle can be high (20–40% in some regions), *T. gondii* is rarely associated with reproductive disease or abortion in cattle [80,81]. This pattern—high exposure, minimal clinical disease—is strikingly similar to the *N. caninum*-buffalo relationship. Understanding why *T. gondii* causes minimal disease in cattle, while *N. caninum* causes devastating abortion, could reveal fundamental principles governing host–pathogen compatibility.

### 6.1. The Zoonotic Enigma: Does Neospora caninum Infect Humans?

The question of whether *N. caninum* infects humans carries profound public health implications and has been investigated for over two decades. Numerous studies have reported antibodies reactive with *N. caninum* antigens in human sera, but the central technical challenge lies in the close phylogenetic relationship between *N. caninum* and *T. gondii*, which share extensive antigenic similarity [82]. A definitive study from Spain by Calero-Bernal et al. [83] analyzed 600 human clinical samples from patients with suspected toxoplasmosis who had tested negative for *T. gondii*. Samples were analyzed by *N. caninum*-specific PCR targeting the Nc5 region. No *N. caninum* DNA was detected in any sample, providing strong evidence against human infection. The current consensus from reference laboratories is that there is no confirmed evidence of *N. caninum* infection in humans [82,83]. This starkly contrasts with *T. gondii*, underscoring the remarkable host specificity of *N. caninum*.

*Connection to the paradox*: The absence of human infection demonstrates that host range is actively restricted by immune recognition mechanisms—directly relevant to why cattle and buffalo differ in their response.

### 6.2. Contrasting Host Ranges: What Determines Susceptibility?

Table 2 summarizes key differences between the two parasites, emphasizing patterns of host susceptibility:

This comparison reveals that *T. gondii* infection in cattle parallels *N. caninum* infection in buffalo—both involve high seroprevalence with minimal reproductive pathology. Conversely, *N. caninum* infection in cattle represents the “susceptible” extreme. This suggests that cattle are uniquely susceptible to *N. caninum*-induced abortion, not because of inherent vulnerability to all apicomplexans, but because of species-specific immune incompatibility with this particular parasite.

### 6.3. Mechanistic Basis: Differential Immune Recognition

Studies in murine models have provided important mechanistic insights. Coombs et al. [58,80] demonstrated that *N. caninum* triggers rapid IFN-β and pro-inflammatory cytokine responses in murine macrophages, while *T. gondii* actively suppresses early innate immunity through secreted effectors (GRA15, ROP16) that modulate host NF-κB and STAT signaling pathways [84]. *N. caninum* appears to lack functional orthologs of these immune evasion factors, rendering it “visible” to the murine immune system.

**Figure 2:**
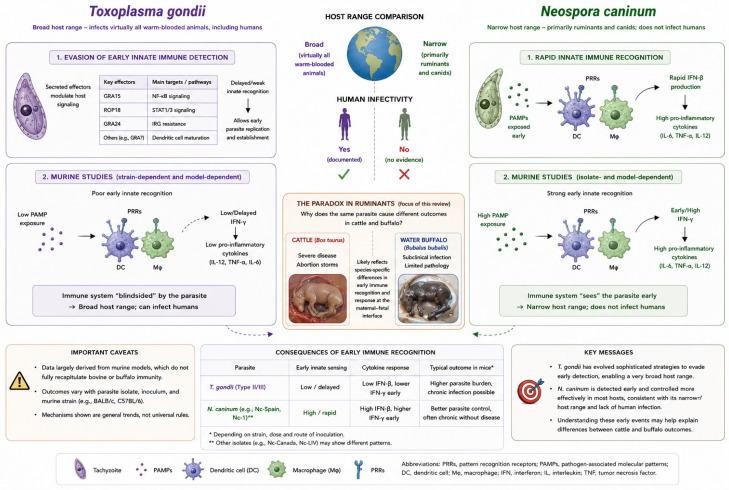
Comparative schematic of host range, innate immune recognition, and species-specific outcomes in *Toxoplasma gondii* and *Neospora caninum* infections. The figure summarizes the contrasting biological and immunological features of the two closely related apicomplexan parasites, *Toxoplasma gondii* (**left panels**) and *Neospora caninum* (**right panels**), with emphasis on differences in host range, innate immune recognition, and implications for disease outcome. *T. gondii* infects a very broad range of warm-blooded hosts, including humans, and possesses multiple secreted effectors (e.g., GRA15, ROP16, ROP18) that modulate host signaling pathways involved in innate immune activation. In contrast, *N. caninum* has a narrower host range, primarily involving ruminants and canids, and no confirmed human infection has been demonstrated. Experimental murine studies indicate that *N. caninum* is generally recognized more rapidly by innate immune pathways, resulting in earlier IFN-β and pro-inflammatory cytokine responses compared with *T. gondii*. The central panel highlights the “ruminant paradox” addressed in this review: despite its narrow host range, *N. caninum* causes severe reproductive disease and abortion in cattle (*Bos taurus*) while usually establishing subclinical persistent infection with limited pathology in water buffalo (*Bubalus bubalis*). The figure proposes that these divergent outcomes may reflect species-specific differences in early immune sensing and regulation at the maternal–fetal interface. Importantly, the mechanisms illustrated derive largely from experimental murine models and should not be directly extrapolated to cattle or buffalo without caution. Host–parasite interactions may vary substantially depending on parasite isolate, inoculum dose, route of infection, experimental model, and host genetic background. Therefore, the pathways shown represent generalized conceptual trends rather than universal mechanisms. Abbreviations: PRRs, pattern recognition receptors; PAMPs, pathogen-associated molecular patterns; DC, dendritic cell; Mφ, macrophage; IFN, interferon; IL, interleukin; TNF-α, tumor necrosis factor-α.

Extrapolating this framework to cattle and buffalo: perhaps *N. caninum* is similarly “visible” to the bovine immune system, triggering inflammatory responses that, while intended to control the parasite, cause collateral damage at the maternal–fetal interface. Conversely, buffalo may have evolved regulatory mechanisms to achieve parasite control without immunopathology—analogous to how cattle control *T. gondii* infection without aborting (Figure 2). This framework transforms the paradox from a descriptive observation into a hypothesis-driven research agenda.

**Important caveat:** The murine model findings described above require direct experimental validation in cattle and buffalo. Immune responses differ substantially between rodents and ruminants, with notable differences in Toll-like receptor expression patterns, cytokine signaling kinetics, and placental structure. Extrapolation of mechanistic conclusions from mice to cattle—and especially to buffalo—must be approached with appropriate caution. The value of the murine studies lies in generating testable hypotheses, not in providing definitive answers about ruminant pathophysiology.

## 7. Future Directions and Conclusions

### 7.1. Future Research Roadmap

Based on the framework developed in this review, we propose a comparative research agenda to translate the buffalo’s natural tolerance into practical interventions for cattle.

Priority 1: *Comparative transcriptomics at the maternal–fetal interface*. Single-cell RNA sequencing of placentomes from infected cattle and buffaloes could reveal cell-type-specific transcriptional responses that distinguish the tolerant host.
Priority 2: *Functional assays of innate immune cells*. Isolate and compare dendritic cells and macrophages from both species to determine whether buffalo phagocytes control *N. caninum* replication more efficiently or produce different cytokine profiles.
Priority 3: *Genome-wide association studies in buffaloes*. Large, well-phenotyped cohorts could identify protective alleles in buffalo populations, using the recently developed buffalo SNP array [85].
Priority 4: *Isolation and characterization of buffalo-derived N. caninum isolates*. No isolate has yet been characterized from water buffalo. This represents a critical gap that must be addressed to determine whether parasite adaptation contributes to the paradox.
Priority 5: *Targeted immunomodulation in cattle*. If buffalo-specific protective pathways are identified, explore whether they can be therapeutically induced in cattle through novel adjuvants or immunomodulatory drugs.

### 7.2. Conclusions

The path to defeating neosporosis in cattle does not lie solely in the study of the disease itself. For three decades, research has focused on the susceptible bovine host—characterizing its pathology, measuring its economic losses, and attempting to achieve protection through vaccination. These efforts, while scientifically valuable, have not delivered transformative control strategies.

The water buffalo offers a different path. In this tolerant host, *N. caninum* establishes a persistent infection without triggering the inflammatory cascade that proves fatal to the bovine fetus. The parasite transmits across generations, yet the host reproduces successfully. This is not resistance in the sense of sterile immunity—buffaloes remain infected—but tolerance: the ability to limit pathology while accepting persistent colonization.

Understanding this natural peace treaty between *N. caninum* and the buffalo could revolutionize neosporosis control. If we can identify the immunological and molecular mechanisms that enable tolerance in buffaloes, we can envision interventions that induce similar states in cattle—not vaccines that aim for sterile immunity (a goal that has proven elusive), but immunomodulatory strategies that shift the host–parasite relationship from pathology toward equilibrium. The paradox with which we began—the silent infection in buffaloes versus epidemic abortion outbreak in cattle—thus becomes not an anomaly to be explained away, but a window into fundamental principles of host–pathogen compatibility. By investigating this paradox, we can move from managing a disease to unlocking a state of equilibrium, transforming the future of neosporosis control worldwide.

## Figures and Tables

**Table 1:** Representative *Neospora caninum* isolates and model-dependent virulence interpretation.

Isolate/Designation	Origin	Virulence Profile	Model-Dependent Interpretation
Nc-1/NC-1	Dog, USA; historical reference isolate	Moderate to model-dependent; fetal death depends on dose, route, and gestational stage	Do not label categorically as highly virulent [18,42]
Nc-Liverpool	Cattle, UK	High virulence in several experimental bovine models	Useful comparator for early-gestation fetal death studies [42]
Nc-Spain7	Spain; bovine origin	High virulence	Induces fetal death, placental parasite burden, and lesions in bovine models [43,44,45]
Nc-Spain1H	Spain; congenitally infected asymptomatic calf	Low virulence/naturally attenuated	Delayed or limited placental infection; evaluated as live-vaccine candidate [44,45,50]
Nc-Spain8	Spain; bovine origin	Low-to-moderate or less rapidly pathogenic under specific conditions	Spanish isolates cannot be treated as a single virulence category [43]
Nc-Nowra	Australia; congenitally infected calf	Low virulence/attenuated	Potential vaccine candidate; distinct from Spanish isolates [48]
Argentine isolates	Argentina; bovine fetal or calf origins	Not a single uniform isolate category	Exact isolate designation and experimental context are required [49]

Note: No *N. caninum* isolate has yet been characterized from water buffalo. Isolation from naturally infected buffalo is a priority for future research.

**Table 2:** Comparative features of *Neospora caninum* and *Toxoplasma gondii*.

Feature	*Neospora caninum*	*Toxoplasma gondii*
Definitive hosts	Canids [11,12]	Felids [80]
Main veterinary reproductive impact	Major abortifacient in cattle [1,2]	Major abortifacient, especially in small ruminants; cattle often show limited clinical disease [80,81]
Human infection	No confirmed human infection by molecular evidence [82,83]	Established zoonosis [81]
Cattle-buffalo relevance	Cattle: susceptible reproductive outcome; buffalo: tolerant phenotype under many field conditions [3,17,18]	Cattle infection with limited disease offers an analogy for host tolerance [81]
Use of murine data	Useful for mechanistic hypotheses but not direct cattle/buffalo proof [59]	Useful for effector biology and immune-evasion comparisons [84]

## Data Availability

No new data were created during this study. Data sharing is not applicable to this article.

## Supplementary Materials

The following supporting information can be downloaded at: https://www.mdpi.com/article/10.3390/microorganisms14061329/s1, Supplementary Materials S1 (The Wildlife Connection—*Neospora caninum* in Sylvatic Cycles).

## Abbreviations

The following abbreviations are used in this manuscript:

IFN-γ	Interferon-gamma
IL	Interleukin
ELISA	Enzyme-Linked Immunosorbent Assay
MICs	Microneme proteins
MAR	Microneme adhesive repeat
ROPs	Rhoptry proteins
PVM	Parasitophorous vacuole membrane
GRAs	Dense granule proteins
CRISPR-Cas9	Clustered regularly interspaced palindromic repeats
PV	Parasitophorous vacuole
NcMYR1	*N. caninum* c-Myc regulatory protein
TLRs	Toll-like receptors
GWAS	Genome-wide association studies
IFAT	Indirect fluorescent antibody test
Tregs	Regulatory T cells
CDC	Center for Diseases Control
AMA1	Apical Membrane Antigen 1
STAT	Signal transducer and activator of transcription
scRNA-seq	Single-cell RNA sequencing
