# Supplementary material for: The Neospora caninum Paradox: Comparative Biology of Cattle and Water Buffalo Reveals Pathways to Control Bovine Neosporosis
Source: Microorganisms. 2026 Jun 13;14(6):1329. doi: 10.3390/microorganisms14061329 (PMC13303815; doi:10.3390/microorganisms14061329)
Supplement: Supplementary file 1 [file microorganisms-14-01329-s001.zip › microorganisms-4286457-supplementary.pdf]

Supplementary Materials S1. The Wildlife Connection – *Neospora caninum* in Sylvatic Cycles

Note: This section has been moved from the main manuscript to supplementary materials to maintain focus on the central cattle-buffalo comparison.

Wild Canids as Definitive Hosts

Neospora caninum circulates within complex ecological communities involving domestic animals, wildlife, and shared environments. Wild canids—wolves (*Canis lupus*), coyotes (*Canis latrans*), and foxes (*Vulpes vulpes*)—serve as definitive hosts in natural ecosystems. Gray wolf populations in North America and Europe show high *N. caninum* seroprevalence (up to 60% in some studies), and infected wolves shed oocysts that contaminate habitats shared with livestock [S1]. Coyotes are particularly significant in North America, where their expanding range brings them into contact with cattle operations [12].

Note: Reference [12] refers to the main manuscript reference list (Gondim et al., 2004 – coyotes as definitive hosts).

Wild Ruminants as Intermediate Hosts

White-tailed deer (*Odocoileus virginianus*) in the United States show seroprevalence ranging from 10% to 70% across different regions, and experimental transmission from deer to dogs has been demonstrated [S2]. Infected deer may introduce the parasite into new areas through migration and serve as a reservoir for spillover into livestock.

Rodents as Reservoirs

Small rodents also play a significant, often overlooked role. *N. caninum* DNA and antibodies have been detected in naturally infected house mice (*Mus musculus*), field mice (*Apodemus sylvaticus*), and brown rats (*Rattus norvegicus*), confirming they are natural intermediate hosts [S3,S4]. A 2023 meta-analysis estimated the global prevalence of *N. caninum* in rodents at 5% (95% CI: 2%–9%), with the highest prevalence in Asia (12%; 95% CI: 6%–24%) [S5]. Experimental evidence further indicates that dogs can become infected by consuming infected rodents, directly linking these small mammals to the domestic transmission cycle [S4]. Capybaras (*Hydrochoerus hydrochaeris*) in South America have also been found to carry *N. caninum* DNA, suggesting that a wide range of rodent species may contribute to the parasite's environmental persistence [S6].

Birds as Potential Long-Distance Vectors

Multiple avian species, including chickens, sparrows, and crows, are susceptible to experimental infection and can harbor parasite DNA in their tissues [S7]. Infected birds might transport the parasite over long distances during migration and contaminate livestock environments with infected feces or carcasses scavenged by canids.

Implications for Control

These wildlife connections complicate eradication efforts. Even if *N. caninum* were eliminated from domestic livestock, wildlife reservoirs could reinfect canids, leading to environmental contamination and spillback into cattle. Effective control therefore requires a landscape-level approach integrating livestock management, wildlife surveillance, and canid population management. However, these considerations are beyond the scope of the main review, which focuses on the cattle-buffalo paradox.

Supplementary References (for this document only)

[S1] Dubey, J.P.; Schares, G.; Ortega-Mora, L.M. Epidemiology and control of neosporosis and *Neospora caninum*. *Clin Microbiol Rev* **2007**, *20*, 323–367. (See section on wildlife) doi:10.1128/CMR.00031-06.

[S2] Dubey, J.P.; Hollis, K.; Romand, S.; Thulliez, P.; Kwok, O.C.H.; Hungerford, L.; Anchor, C.; Etter, D. High prevalence of antibodies to *Neospora caninum* in white-tailed deer (*Odocoileus virginianus*). *Int J Parasitol* **1999**, *29*, 1709–1711. doi:10.1016/S0020-7519(99)00127-7.

[S3] Huang, C.C.; Yang, C.H.; Watanabe, Y.; Liao, Y.K.; Ooi, H.K. Finding of *Neospora caninum* in the wild brown rat (*Rattus norvegicus*). *Vet Res* **2004**, *35*, 283–290. doi:10.1051/vetres:2004010.

[S4] Ferroglio, E.; Pasino, M.; Romano, A.; Grande, D.; Pregel, P.; Trisciuoglio, A. Evidence of *Neospora caninum* DNA in wild rodents. *Vet Parasitol* **2007**, *148*, 346–349. doi:10.1016/j.vetpar.2007.06.031.

[S5] Hamzavi, Y.; Salimi, Y.; Ahmadi, M.; Adimi, P.; Falahi, S.; Bozorgomid, A. Global prevalence of *Neospora caninum* in rodents: A systematic review and meta-analysis. *Vet Med Sci* **2023**, *9*, 2192–2200. doi:10.1002/vms3.1196.

[S6] Truppel, J.H.; Reifur, L.; Montiani-Ferreira, F.; Lange, R.R.; de Barros Filho, I.R.; de Oliveira, L.G.S.; de Souza, L.F.; de Oliveira, R.T.S.; de Souza, L.C.P.; Biondo, A.W.; et al. Detection of *Neospora caninum* DNA in capybaras and phylogenetic analysis. *Parasitol Int* **2010**, *59*, 376–379. doi:10.1016/j.parint.2010.05.006.

[S7] Costa, K.S.; Santos, S.L.; Uzêda, R.S.; Pinheiro, A.M.; Almeida, M.A.; Araújo, F.R.; McAllister, M.M.; Gondim, L.F. Chickens (*Gallus domesticus*) are natural intermediate hosts of *Neospora caninum*. *Int J Parasitol* **2008**, *38*, 157–163. doi:10.1016/j.ijpara.2007.10.008.

Note to the editor: The citation [12] in the first section refers to the main manuscript reference list (Gondim et al., 2004). All other citations [S1]–[S7] are provided in this supplementary reference list. This avoids renumbering the main reference list while keeping the supplementary material self-contained and fully citable.
